# Intratracheal Leiomyosarcoma in a 13-Year-Old German Riding Pony Mare

**DOI:** 10.3390/vetsci13080800

**Published:** 2026-08-13

**Authors:** Jennifer Hiekel, Christoph Kühnle, Klaudia Zofia Blaszczyk

**Affiliations:** Pferdepraxis Kühnle GbR, Parkstraße 7, 74532 Ilshofen, Germany; ckuehnle@pferdepraxis-kuehnle.com (C.K.); kblaszczyk@pferdepraxis-kuehnle.com (K.Z.B.)

**Keywords:** equine, leiomyosarcoma, intratracheal, neoplasia

## Abstract

A 13-year-old German Riding Pony mare was referred for evaluation of progressive respiratory noise and mixed dyspnoea, which rapidly deteriorated to respiratory distress. Clinical examination revealed marked rattling inspiratory and expiratory tracheal sounds and severe, rattling, vesicular lung sounds. Arterial blood gas analysis demonstrated moderate hypoxaemia (partial pressure of arterial oxygen 76 mmHg at rest). Upper airway endoscopy identified a pink, cauliflower-like pedunculated mass originating from the dorsal tracheal wall approximately 15 cm cranial to the tracheal bifurcation, causing an estimated 60% luminal obstruction. The mass was successfully removed via transendoscopic electrosurgical excision. However, complete tumour-free margins were not achieved. Histopathological examination revealed the diagnosis of leiomyosarcoma. Following surgery, the mare exhibited rapid clinical improvement. Peri-operative treatment included antimicrobial, anti-inflammatory, and bronchodilatory therapy. A follow-up endoscopy one month after surgery showed adequate healing of the surgical site. A small, vascularized tissue structure within the scar tissue, considered suspicious for residual tumour growth, was identified. However, no significant progression was observed during a follow-up examination seven months postoperatively. This case highlights tracheal leiomyosarcoma as a rare differential diagnosis in horses with respiratory distress and describes transendoscopic electrosurgical excision as a feasible therapeutic option.

## Simple Summary

A 13-year-old German Riding Pony mare developed worsening respiratory noise and breathing difficulty that rapidly progressed to severe respiratory distress. On examination, blood testing showed reduced oxygen levels, and endoscopy of the upper airway revealed a cauliflower-like growth arising from the wall of the trachea, blocking about 60% of the airway. Under sedation, the mass was removed using a minimally invasive, endoscope-guided electrosurgical technique performed directly through the airway, avoiding the need for open surgery. Although the tumour was successfully excised and the airway obstruction resolved, complete tumour-free margins could not be confirmed. Histopathological examination identified the growth as a leiomyosarcoma, a malignant tumour of smooth muscle origin. Following the procedure, the mare recovered quickly and received supportive treatment with antibiotics, anti-inflammatory drugs, and bronchodilators. A follow-up endoscopy one month later showed good healing of the surgical site, though a small area of tissue within the scar raised suspicion of possible residual tumour. However, no further tumour growth was observed at a seven-month recheck. This report describes, for the first time, an intratracheal leiomyosarcoma in a horse and demonstrates that transendoscopic electrosurgical excision can be a feasible treatment option for this rare type of airway tumour.

## 1. Introduction

Leiomyosarcomas are malignant neoplasms, originating from smooth muscle cells. Only one single case report about a benign intratracheal leiomyoma [1] has been described in horses. To date, no cases of intratracheal leiomyosarcomas have been described in horses. Respiratory neoplasias are generally uncommon in horses [2], and leiomyosarcomas are considered rare within the respiratory tract across species [3,4].

In horses, leiomyosarcomas have been reported in various other anatomical locations, including the testes [5], lungs [6], guttural pouches [7], gastrointestinal tract [8,9], tongue [10] and ocular tissue [11]. In humans, these tumours most commonly arise in the uterus or gastrointestinal tract, although sporadic cases involving the respiratory system have also been described [12,13,14].

To the authors’ knowledge, this report describes the first case of a deep intratracheal leiomyosarcoma in a 13-year-old pony mare.

## 2. Case Report

A 13-year-old German Riding Pony mare was referred in August 2025 for further evaluation of progressive inspiratory and expiratory respiratory noise associated with mixed dyspnoea. The mare had previously been diagnosed by another veterinarian with a suspected tracheal cyst based on endoscopic findings and had been treated empirically for suspected equine asthma. Initially, no increased respiratory effort was observed; however, the mare subsequently developed exercise intolerance, which progressively worsened over time and prompted referral for further diagnostic evaluation.

### 2.1. Clinical Findings

At presentation, the mare was initially bright and alert. However, within the first minutes of the examination, she suddenly developed inspiratory and expiratory respiratory sounds. Her condition rapidly deteriorated into respiratory distress, characterised by flared nostrils, pronounced abdominal breathing effort, and generalised sweating. Vital parameters were within normal ranges, except for the respiratory rate (heart rate 40 beats per minute, respiratory rate 28 breaths per minute, rectal temperature 37.8 °C). Mucous membranes were pale pink and moist, and the capillary refill time was <2 s.

Thoracic auscultation revealed normal cardiac sounds; however, respiratory sounds were abnormal and were characterised by a severe tracheal rattle and increased vesicular sounds. Increased vesicular sounds were noted in all pulmonary fields and were accompanied by marked tracheal rattle with increased inspiratory and expiratory respiratory sounds over the trachea.

### 2.2. Further Diagnostics

**Figure 1:**
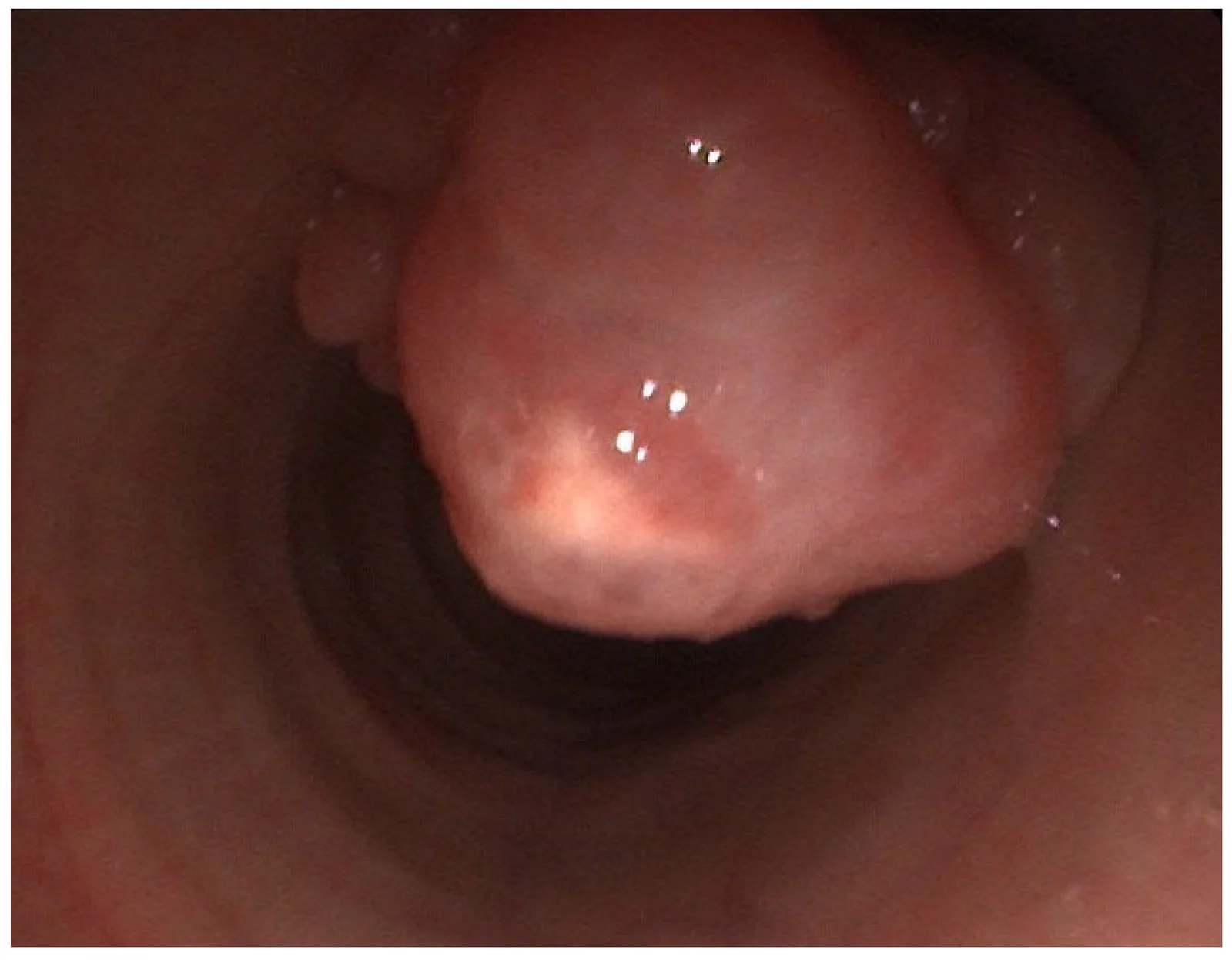
Endoscopic picture of the cauliflower-like, pedunculated mass (approximately 40 × 30 × 25 mm).

An arterial blood sample was collected from the common carotid artery for blood gas analysis (ScilVet, EDAN i15VT), which revealed a resting partial pressure of oxygen (pO_2_) of 76 mmHg (reference range: 90–110 mmHg). All remaining assessed parameters were within normal reference intervals. Furthermore, an upper airway endoscopy (SHINOVA^®^, GASTRIX 85 V, 1500 mm, Shanghai, China) was performed, under mild sedation of the horse by a combination of xylazine (Xylavet^®^ 100 mg/mL, cp pharma, 0.1 mg/kg, i.v.), detomidine (Domidin^®^ 10 mg/mL, cp-pharma, 0.01 mg/kg, i.v.), acepromazine (Tranquisol^®^ P 10 mg/mL, cp-pharma, 0.01 mg/kg, i.v.) and butorphanol (Torphadine^®^ 10 mg/mL, cp-pharma, Burgdorf, Germany, 0.01 mg/kg, i.v.). The larynx appeared within normal limits. The pharyngeal mucosa was mildly hyperaemic, and moderate viscous mucus was visible in the pharynx and trachea (tracheal mucus score 2/5, according to Gerber et al. [15]). Additionally, approximately 15 cm cranial to the bifurcation, a pink, cauliflower-like pedunculated mass (approximately 40 × 30 × 25 mm) was identified arising from the dorsal tracheal wall and protruding into the lumen, resulting in an estimated 60% lumen obstruction (Figure 1).

A transendoscopic tracheal wash sample (TBS) was obtained at the most distal accessible point of the trachea, cranial to the visible mass and sent for cytology (EquiZyt UG laboratories, Steinhöring, Germany). At this stage of the examination, the lower respiratory tract distal to the mass could not be evaluated, as the lesion prevented passage and manipulation resulted in acute respiratory deterioration with respiratory distress. Consequently, bronchoalveolar lavage (BAL) was not performed, as management of the obstructing mass was prioritised. However, BAL remained indicated for subsequent evaluation of suspected concurrent equine asthma and chronic airway irritation secondary to the mass.

The cellularity of the TBS sample was consistent with a neutrophilic inflammation (55% neutrophils (10–30%)) with intra- and extracellular bacteria.

**Figure 2:**
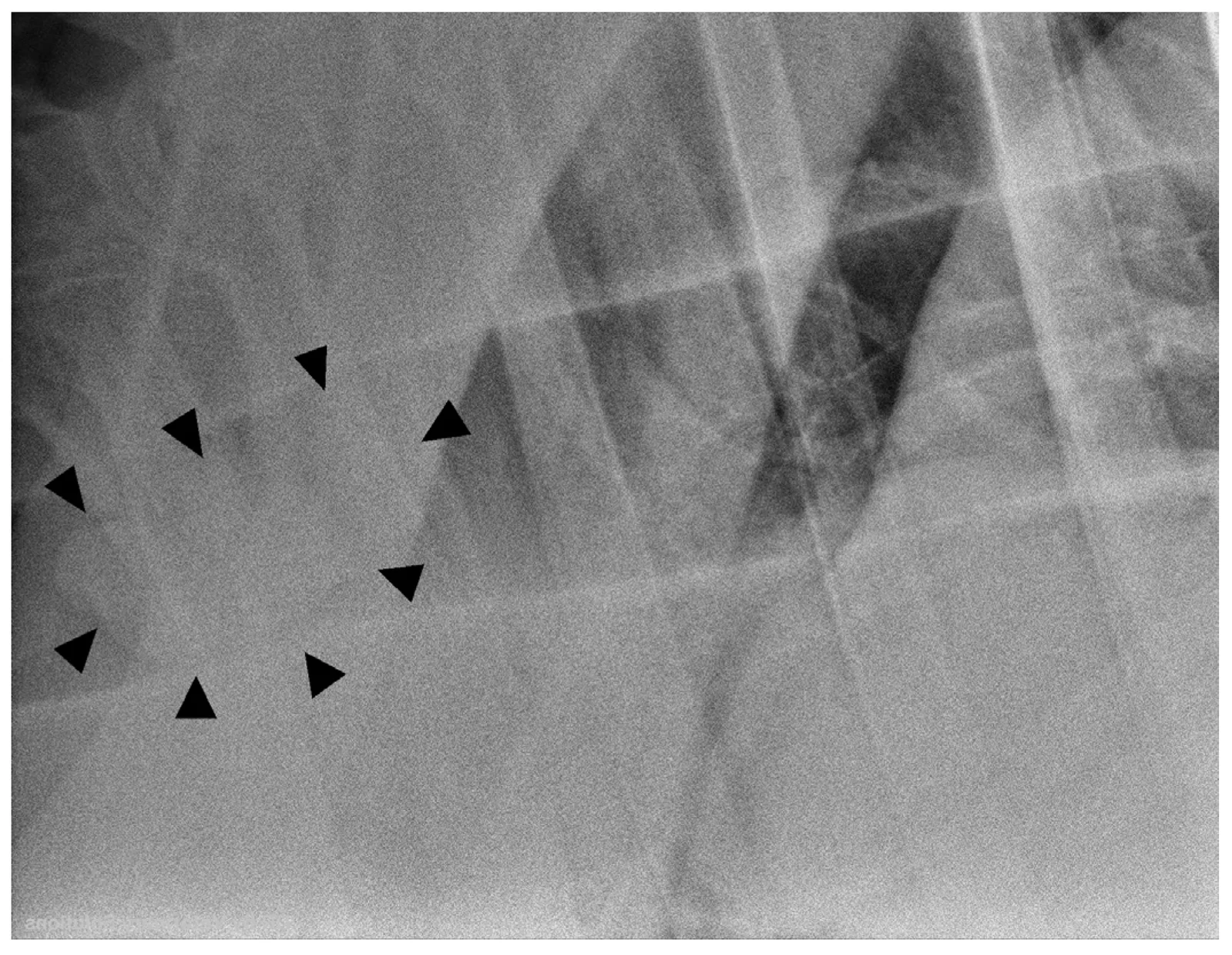
A pre-surgical radiograph of the trachea (with the caudal end to the right and the proximal end at the top) demonstrates a well-defined, round soft-tissue mass within the tracheal lumen at the level of the thoracic inlet, indicated by the black arrowheads.

A radiographic evaluation revealed a well-circumscribed, round mass of soft-tissue opacity within the tracheal lumen at the level of the thoracic inlet (Figure 2).

The presumptive diagnosis included an intratracheal neoplasia or granuloma, as well as an acute bacterial respiratory infection, with a potentially underlying equine asthma.

### 2.3. Surgery

Surgical excision of the tracheal mass was performed promptly on the day of admission under standing sedation. Presurgical haematological and serum biochemical parameters (ScilVet Element HT 5 and element DC) were within normal limits.

An intravenous catheter (MILACATH^®^-EXTENDED USE, 12 Ga × 13 cm, radiopaque Polyurethan) was placed in the left jugular vein. The mare received perioperative non-steroidal anti-inflammatory and antimicrobial treatment, including flunixin meglumine (FLUMEG NOVA 5%, Serumwerk Bernburg AG, Burgdorf, Germany, 1.1 mg/kg i.v.), amoxicillin (Belamox^®^ 200 mg/mL, bela-pharm, Vechta, Germany, 10 mg/kg, i.v.), and gentamicin (Genta^®^ 100 mg/mL, cp pharma, 9 mg/kg i.v.).

Sedation was induced by administration of a bolus combination of xylazine (0.1 mg/kg, i.v.), detomidine (0.01 mg/kg, i.v.), acepromazine (0.01 mg/kg, i.v.) and butorphanol (0.01 mg/kg, i.v.). Furthermore, it was maintained by applying a constant rate infusion of an alpha2-agonist (24 mg detomidine) together with an opioid-agonist-antagonist (10 mg butorphanol) diluted in 500 mL isotonic sodium-chloride solution (0.9%, DELTAMEDICA, Reutlingen, Germany) and titrated to effect.

For monopolar electrosurgery, a patient return electrode was placed on the horse’s neck.

The endoscope (SHINOVA^®^, GASTRIX 85 V, 1500 mm) was introduced through the right nostril into the trachea, and the surgical site was desensitised by topical application of mepivacaine (Mepidor^®^, 20 mg/mL, Richter Pharma AG, Wels, Austria; 200 mg topical) via the working channel of the endoscope. Subsequently, a second flexible endoscope (video endoscope Eickview 150 E, 8 × 1500 mm, Eickemeyer KG, Tuttlingen, Germany) was introduced through the left nostril alongside the first endoscope. As the endoscopes partially occupied both nostrils during the procedure, airflow was further compromised in the already dyspnoeic mare. Therefore, the intervention had to be performed efficiently to minimise additional respiratory compromise. Despite this, the mare tolerated the procedure well. A snare was advanced through the working channel of the second endoscope, positioned around the widest portion of the mass, and securely tightened to ensure continuous control of the mass during the procedure and to prevent dislodgement into the bronchi. Through the working channel of the first endoscope, a flexible electrosurgical instrument (ERBE ICC 200, Erbe Elektromedizin GmbH, Tübingen, Germany) was inserted and used to dissect the mass as close as possible to the tracheal wall. Particular care was taken to preserve the tracheal cartilage. The positioning of the two endoscopes enabled simultaneous use of the snare and the electrosurgical instrument. In addition, the availability of two different viewing angles improved visualisation and facilitated the intervention. Despite concurrent cauterisation of the tissue during dissection, mild haemorrhage occurred throughout the procedure, resulting in intermittent impairment of visualisation. However, following complete transection at the base of the mass and local cauterisation of the surgical bed, haemorrhage ceased entirely. The mass was then carefully retrieved under endoscopic guidance through the left ventral nasal meatus and nostril while still secured with the snare.

The excised mass was submitted in toto for histopathological examination (IDEXX GmbH laboratories, Kornwestheim, Germany).

### 2.4. Postoperative Treatment

The mare was hospitalised for four days for postoperative treatment and surveillance.

Due to the profound vascularisation of the mass and the associated haemorrhage, aspiration of blood was considered likely. Therefore, intravenous antimicrobial therapy (amoxicillin 10 mg/kg BID and gentamicin 9 mg/kg SID) as well as non-steroidal anti-inflammatory treatment (flunixin meglumine 1.1 mg/kg, i.v. BID) were continued during hospitalisation. Considering an underlying equine asthma, treatment with clenbuterol and dembrexine (Venti Plus^®^, clenbuterol hydrochloride and dembrexine hydrochloride, Boehringer Ingelheim, Ingelheim am Rhein, Germany, 50 mg/kg bodyweight, BID p.o.) was initiated and maintained until further diagnostic evaluation. In addition, the mare received macrogol (MACROGOL AL, 13.7 g, ALIUD PHARMA, Laichingen, Germany, 4 sachets BID p.o., off-label) for the prophylaxis of constipation.

Directly after surgery, the mare showed paroxysmal coughing when being fed with steamed hay on the floor; therefore, she only received mashes and soaked hay cubes on the first two days postoperatively. On the third day, no further coughing was observed. Therefore, steamed hay was reintroduced without any subsequent problems.

Vital parameters, as well as breathing pattern, remained within normal limits, and no abnormal inspiratory or expiratory sounds were noted.

On days one and two post-surgery, an ultrasonographic evaluation of the thorax showed mild comet-tail artefacts on both sides of the lungs. A small volume of hypoechogenic pleural effusion and mild pulmonary consolidation were detected in the left cranioventral thorax, both of which had largely resolved by postoperative day four.

Follow-up endoscopic examinations were performed on day one and four post-surgery. No fluid or haemorrhage was noted at any time; the surgical site was hyperaemic at first, and on day four, a small plaque had formed.

On the fourth day post-operatively, the serum amyloid A (SAA) was 37 µg/mL (VMRD equine SAA test; reference interval 0–20 µg/mL).

For continued treatment at home, medication was transitioned to oral trimethoprim sulphonamide (Sulfadimethoxin + Trimethoprim 50%, 417 mg/g + 83 mg/g, Serumwerk Bernburg, 20 mg/kg BID p.o.) and meloxicam (Melosus^®^, 15 mg/mL, CP-Pharma Handelsgesellschaft mbH, Burgdorf, Germany; 0.6 mg/kg SID p.o.). Clenbuterol and dembrexine were continued orally for 3.5 weeks, and the owner was educated to seek a dust-reduced environment for the mare.

Histologic examination of the neoplasia showed a nonencapsulated, diffuse infiltrative neoplasia composed of moderately dense spindle cells forming irregular bundles and clusters within a large amount of fibrillar, eosinophilic, extracellular matrix. The cytoplasm was eosinophilic with indistinguishable cell borders. Nuclei were spindle- to cigar-shaped, with granular chromatin and indistinct nucleoli. Anisocytosis and -karyosis were graded mild to moderate, and the mitotic count was 2.37 mm^2^:1. A mild inflammatory response, composed of neutrophils and lymphocytes, was observed. The Elastica van Gieson stain was positive; predominantly smooth muscle fibres were detected. Based on histological evidence, a diagnosis of tracheal well-differentiated leiomyosarcoma was made, revealing incomplete surgical margins. Immunohistochemical evaluation demonstrated strong desmin immunoreactivity in the tumour cells, indicating myogenic differentiation.

### 2.5. Follow-Up

Four weeks after the procedure, the mare was presented for the first follow-up examination. The clinical examination was within normal limits, and the owners reported no further dyspnoea. Thoracic ultrasonography revealed mild bilateral comet-tail artefacts throughout the entire lung field, with a greater extent on the right side than on the left. Additionally, mild pulmonary consolidation was detected in the cranial right lung. No free fluid was noted, but the pleura was mildly non-homogeneous. No extratracheal mass growth was identified on ultrasonographic examination of the trachea.

**Figure 3:**
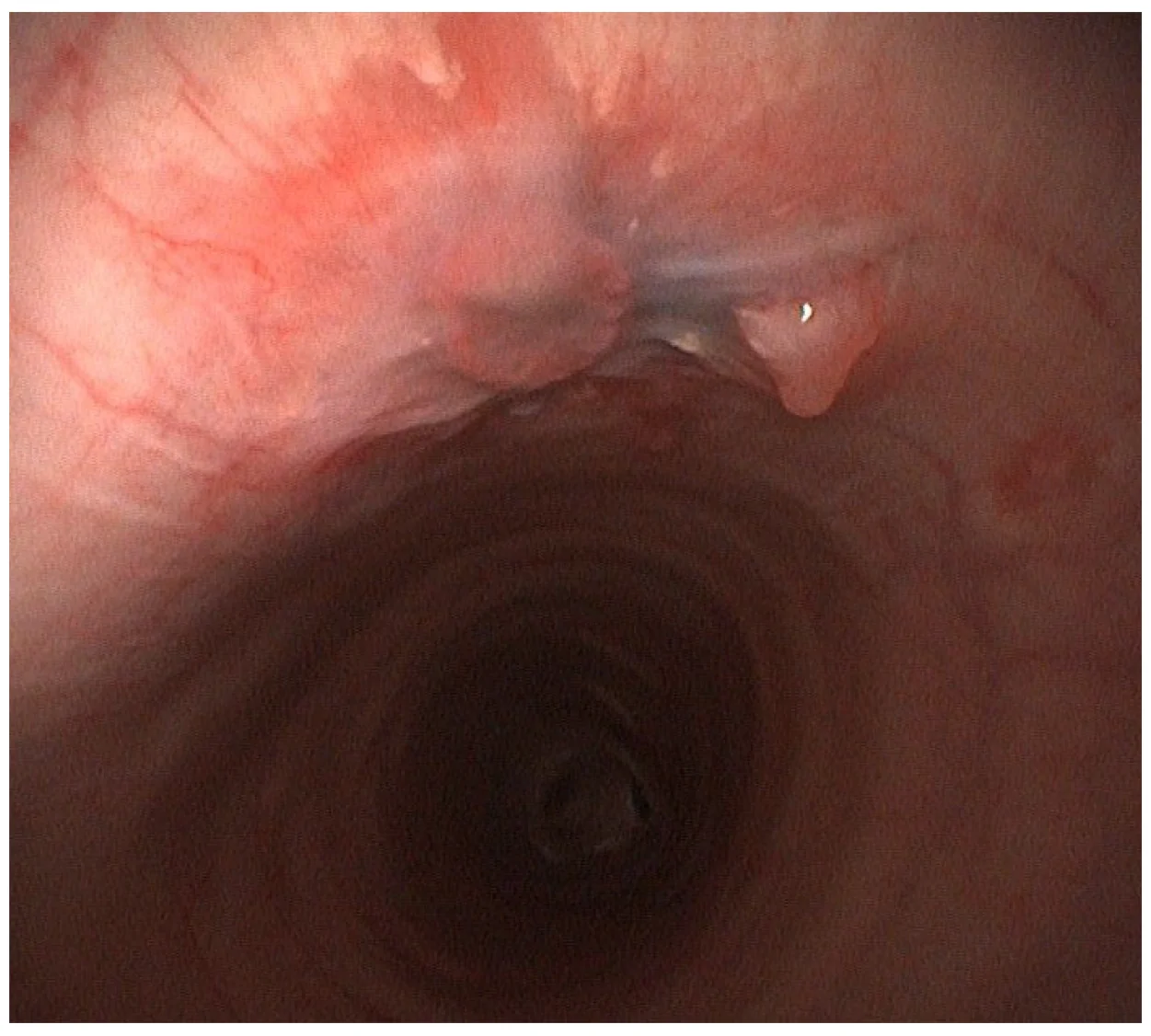
Endoscopic picture of the surgical site four weeks post-surgery.

In a follow-up endoscopy of the upper airway, a mild follicular pharyngitis, as well as viscous mucus in the trachea, was noted (tracheal mucus score 3/5 [15]). The surgical site healed well, and only mild scar tissue formation was seen. Within the scar formation, a vascularized tissue structure of uncertain origin, considered suspicious for residual tumour tissue, was identified. However, no tumour re-growth was noted at that time (Figure 3).

A TBS as well as a BAL sample was collected at this time and sent to an external laboratory for cytology (EquiZyt UG laboratories, Steinhöring, Germany). Cytological findings were indicative of mild to moderate equine asthma or reactive bronchitis (neutrophils: TBS 41% (10–30), BAL 15% (0–8%); eosinophils: TBS 5% (0%); mast cells: BAL 6% (0–5%)).

Therefore, after submitting baseline adrenocorticotrophic hormone (ACTH) and insulin concentrations to an external reference laboratory (Laboklin GmbH & Co KG laboratories, Bad Kissingen, Germany), which were within normal limits (ACTH < 5.00 pg/mL (<30 pg/mL); insulin 11.3 µU/mL (<15 µU/mL)), oral prednisolone (Equipred, 50 mg, cp pharma, 1 mg/kg p.o. SID, for 2 weeks) therapy was started, followed by long-term inhalation with budesonide (Pulmicort, 1 mg/2 ml, Astra Zeneca, Sweden, 1–2 mg/d p. i.).

The pO_2_ had normalised (117 mmHg (90–110 mmHg)), and radiographs of the lungs did not show any abnormalities.

Six months later, a second follow-up examination was performed. The owners reported that the mare was fully returned to leisure-level exercise and was no longer receiving any medication. Occasional coughing was observed only once at the onset of trotting exercise, while no further respiratory signs were reported. Cardiac auscultation was unremarkable. However, mildly increased vesicular lung sounds were detected bilaterally, more pronounced on the right side. Vital parameters remained within normal limits.

Follow-up arterial blood gas analysis at rest revealed a pO_2_ of 83 mmHg (90–110 mmHg) at rest.

**Figure 4:**
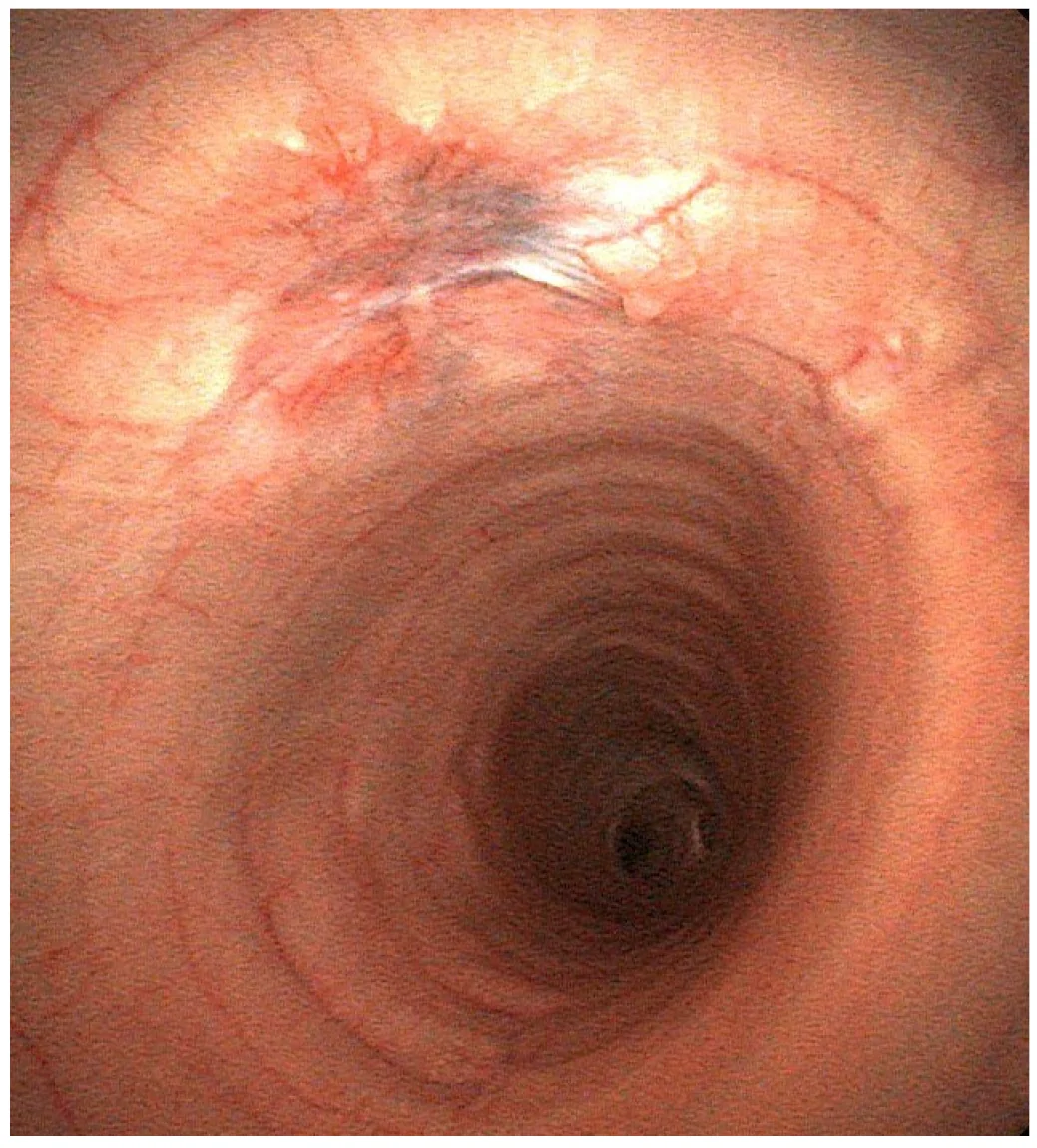
Endoscopic picture of the surgical site seven months post-surgery.

Upper airway endoscopy revealed a moderate amount of white, highly viscous tracheal mucus (tracheal mucus score 3/5 [15]). Within the previously described hyperaemic, smooth, and well vascularized scar tissue, the vascularized structure of uncertain origin, previously considered suspicious for residual tumour tissue, showed only minimal progression in size (Figure 4).

A repeated BAL was performed and submitted to an external laboratory (EquiZyt laboratories, Steinhöring, Germany) for cytological evaluation. Mild to moderate equine asthma was confirmed based on an increased neutrophil proportion (17% (0–8%)). Consequently, treatment with dembrexine (Sputolysin ^®^, dembrexinehydrochloride 5 mg/g, 0.3 mg/kg body weight BID, p.o., Boehringer Ingelheim) was initiated for two weeks, after which the horse showed marked clinical improvement.

Continued follow-up examinations at six-month intervals were recommended.

Generative artificial intelligence was used exclusively for language editing, including grammar, style, and readability. It was not used to generate scientific content, analyse data, interpret results, or influence the conclusions of this manuscript. The authors remain solely responsible for all scientific content and the final version of the manuscript.

## 3. Discussion

Similar to reports of tracheal leiomyosarcoma in humans, the horse initially presented with non-specific clinical signs such as dyspnoea, cough and abnormal respiratory noise [4]. Initial treatment was directed towards more common conditions, such as equine asthma, until further diagnostics were initiated, contributing to a delayed diagnosis [3]. A BAL and TBS analysis, performed four weeks postoperatively, indicated concurrent mild to moderate equine asthma in addition to the tracheal neoplasia. The coexistence of these conditions may complicate an early accurate diagnosis and subsequent clinical monitoring, as clinical signs are overlapping.

Therapeutically, surgical excision of the tumour was considered the treatment of choice, as the lumen of the trachea was already markedly occluded. Resection of the mass was therefore required to secure the airway and reduce the risk of progression or recurrence [14].

Excision of the neoplastic lesion at this anatomical location was considered technically challenging, as several surgical approaches are not feasible within the trachea. Potential complications, such as haemorrhage, aspiration with subsequent aspiration pneumonia, surgical site infection or tissue necrosis were discussed.

Emergency tracheotomy would not have been possible, due to the fact that the lesion was located too far caudal in the trachea, thereby posing a substantial risk of catastrophic decompensation. 

Conventional segmental resection with end-to-end anastomosis of the trachea was discussed, but would have been a very invasive procedure, as the trachea would have had to be exteriorised to facilitate adequate surgical site preparation. This would have likely resulted in considerable traction on the lungs and carried a high risk of haemorrhage. Afterwards, excessive tension at the anastomotic site (for example, when the horse would have stretched its neck) would have increased the likelihood of dehiscence [16]. Postoperative tracheal stenosis was also considered a significant potential complication [16].

Laser excision and ablation were ruled out due to the substantial risk of thermal injury to the tracheal cartilage in proximity and subsequent necrosis [17]. Endoscopic local excision using an electrotome was ultimately selected, as this approach allowed minimally invasive access while providing simultaneous haemostasis. A major limitation of this approach is that it predominantly enables tumour reduction. While complete tumour excision may be possible, the likelihood of achieving histologically tumour-free margins is generally lower compared with segmental tracheal resection, which permits en bloc resection with wider tissue margins.

Another major intraoperative concern was inadvertent dislodgement of the tumour into the distal trachea or bronchi during resection, which could have resulted in acute airway obstruction. This risk was minimised by securing the mass with a snare introduced via a second endoscope throughout the procedure.

A notable side effect of electrotomy is the generation of surgical smoke during tissue removal. Therefore, butorphanol was included in the sedation protocol not only for its analgesic effects but also for its antitussive properties. Furthermore, local airway irritation associated with exposure to electrosurgical plume may have contributed to the postoperative cough observed in this case, as it contains ultrafine particles and irritant compounds that may affect the respiratory mucosa [18].

Complete surgical resection is considered the gold standard in human medicine [12,13,14]. Systemic chemotherapy may be employed as an adjunctive treatment or in cases where complete resection is not feasible. However, its efficacy remains variable and is subject to ongoing debate [12,13]. Radiation therapy has also been described as a means of reducing the risk of local recurrence, not of metastasis [12,13]. In the present case, adjuvant chemotherapy or radiotherapy were discussed with the owner. However, no chemotherapeutic treatment had been described in equine cases previously, and the use of these modalities in equine patients is further constrained by practical, economic, and logistical constraints.

The administration of prednisolone was considered in this case, primarily in the context of concurrent equine asthma and its long-term management. Beyond its established anti-inflammatory effects, glucocorticoids have been suggested to exert modulatory effects on tumour cell proliferation in experimental leiomyosarcoma models. However, the clinical relevance of these findings remains uncertain [19]. To support therapeutic decision-making and assess the potential risks associated with glucocorticoid administration, baseline insulin and ACTH concentrations were measured. Dynamic insulin testing was advised to identify an underlying endocrine dysfunction, which would have raised additional concerns.

Regular follow-up examinations, including endoscopic evaluations, as well as advanced imaging of the abdomen and thorax, were recommended to monitor local recurrence or metastatic spread. To date, metastasis has not been identified, which is consistent with reports suggesting that leiomyosarcomas metastasise slowly [8,12,13].

A prognosis is difficult to determine, but given the malignancy of the neoplasia, the non-tumour-free margins, as well as the local invasive growth, the risk of recurrence remains high. However, as the surgical site appeared to be healing well on gross examination, surgery to achieve tumour-cell-free margins was held off at this stage. Furthermore, biopsy sampling of the small, vascularized tissue structure of uncertain origin detected during follow-up endoscopies was withheld due to concerns regarding potential stimulation of recurrent tissue growth.

## 4. Conclusions

This case report highlights tracheal leiomyosarcoma as an uncommon but clinically relevant differential diagnosis in horses presenting with respiratory distress. Endoscopic local excision using an electrotome proved to be a feasible therapeutic approach and resulted in substantial clinical improvement. However, complete tumour-free surgical margins could not be achieved using this technique. Therefore, continued long-term follow-up is required to assess potential tumour recurrence and long-term outcome.

Further studies are needed to improve the understanding of tumour progression and establish treatment recommendations.

## Data Availability

The original contributions presented in this study are included in the article. Further inquiries can be directed to the corresponding author.
